# A Novel Sweetpotato Transcription Factor Gene *IbMYB116* Enhances Drought Tolerance in Transgenic *Arabidopsis*

**DOI:** 10.3389/fpls.2019.01025

**Published:** 2019-08-15

**Authors:** Yuanyuan Zhou, Hong Zhu, Shaozhen He, Hong Zhai, Ning Zhao, Shihan Xing, Zihao Wei, Qingchang Liu

**Affiliations:** ^1^Key Laboratory of Sweetpotato Biology and Biotechnology, Ministry of Agriculture/Beijing Key Laboratory of Crop Genetic Improvement/Laboratory of Crop Heterosis and Utilization, Ministry of Education, College of Agronomy & Biotechnology, China Agricultural University, Beijing, China; ^2^College of Agronomy, Qingdao Agricultural University, Qingdao, China

**Keywords:** sweetpotato, *IbMYB116*, *Arabidopsis*, drought tolerance, JA signaling pathway

## Abstract

Several members of the MYB transcription factor family have been found to regulate growth, developmental processes, metabolism, and biotic and abiotic stress responses in plants. However, the role of MYB116 in plants is still unclear. In this study, a MYB transcription factor gene *IbMYB116* was cloned and characterized from the sweetpotato [*Ipomoea batatas* (L.) Lam.] line Xushu55-2, a line that is considered to be drought resistant. We show here that IbMYB116 is a nuclear protein and that it possesses a transactivation domain at the C terminus. This gene exhibited a high expression level in the leaf tissues of Xushu55-2 and was strongly induced by PEG6000 and methyl-jasmonate (MeJA). The *IbMYB116*-overexpressing *Arabidopsis* plants showed significantly enhanced drought tolerance, increased MeJA content, and a decreased H_2_O_2_ level under drought stress. The overexpression of *IbMYB116* in *Arabidopsis* systematically upregulated jasmonic acid (JA) biosynthesis genes and activated the JA signaling pathway as well as reactive oxygen species (ROS)-scavenging system genes under drought stress conditions. The overall results suggest that the *IbMYB116* gene might enhance drought tolerance by activating a ROS-scavenging system through the JA signaling pathway in transgenic *Arabidopsis*. These findings reveal, for the first time, the crucial role of *IbMYB116* in the drought tolerance of plants.

## Introduction

Drought seriously affects the productivity of agricultural crops in the world (Yang et al., 2010; Boyer et al., 2013). Improving the drought tolerance of crops has become important for food security (Tester and Langridge, 2010; Zhu, 2016). Plants adapt to drought stress by developing a variety of mechanisms, including growth and development regulation, osmotic adjustment, ion homeostasis, and detoxification (Bohnert et al., 1995; Zhu, 2002). In response to water deficits, plants increase water uptake by forming long roots to promote their survival (Yu et al., 2008; Wang et al., 2016a). At the phytohormone level, jasmonic acid (JA), abscisic acid (ABA), salicylic acid (SA), and ethylene (ETH) play important roles in plant growth and development as well as protective responses against biotic and abiotic stresses (Fujita et al., 2005; Dong et al., 2014; Vargas et al., 2014; Zhang et al., 2015a; Jin et al., 2016; Xu et al., 2017).

Jasmonates (JAs), including JA, methyl-jasmonate (MeJA), and JA-isoleucine (JA-Ile), are important regulators of plant responses to environmental stresses, such as drought, salt, and ozone exposure (Overmyer et al., 2000; Turner et al., 2002; Lorenzo et al., 2003; Rojo et al., 2003; Wasternack et al., 2006; Dhakarey et al., 2017; Zhang et al., 2017). In JA biosynthesis, plastidial 13-lipoxygenase (LOX), allene oxide synthase (AOS), and allene oxide cyclase (AOC) catalyze linolenic acid to *cis*-12-oxophytodienoic acid (OPDA); OPDA reductase (OPR) further reduces OPDA to 3-oxo-2(2’(Z)-pentenyl)-cyclopentane-1-octanoic acid (OPC-8:0). Subsequently, the carboxyl chain of OPC-8:0 is shortened by three β-oxidation steps, which form JA with the key enzymes CoA ligase 1 (OPCL1), acyl-CoA oxidase (ACOX1, ACOX3), enoyl-CoA hydratase (MFP2), acetyl-CoA acyltransferase (fadA), and acetyl-CoA acyltransferase 1 (ACAA1) (Mueller, 1997; Berger, 2002; Wasternack and Hause, 2013). Furthermore, coronatine-insensitive 1 (COI1), jasmonate ZIM-domain (JAZ) and myelocytomatosis (MYC) proteins are the components of the core JA signaling pathway (Chini et al., 2007; Wasternack and Hause, 2013; Liu and Avramova, 2016). The JA signaling pathway plays a critical role in regulating the response to drought stress in plants (Liu et al., 2015; Ahmad et al., 2016).

In plants, several types of transcription factors (TFs), including MYB/MYC, NAC, bZIP, AP2/ERF, PHD, and WRKY, control biological processes by regulating target genes in response to stresses (Shiriga et al., 2014; Zong et al., 2016; Wang et al., 2017a; Wang et al., 2017b). MYB is a large transcription factor family in higher plants (Riechmann et al., 2000). Based on the number of repeats of the MYB domain, this family is divided into four major classes: 1R-MYB, R2R3-MYB, 3R-MYB, and 4R-MYB. *ZmC1* from *Zea mays* was the first plant *MYB* gene (Paz-Ares et al., 1987). To date, more than 200 MYB TFs have been identified in several plant species, such as *Arabidopsis*, rice, and soybean (Dubos et al., 2010; Aoyagi et al., 2014; Smita et al., 2015). R2R3-MYB TFs have been found to play important roles in plant responses to biotic and abiotic stresses (Dubos et al., 2010). Accumulated evidence has indicated that R2R3-MYB TFs mediate stress-signaling pathways, such as salinity, drought, and extreme temperature (Hajiebrahimi et al., 2017). In *Arabidopsis*, several R2R3-MYB TFs, including AtMYB2, AtMYB15, AtMYB44, AtMYB60, AtMYB61, AtMYB96, and AtMYB102, were involved in drought stress responses (Denekamp and Smeekens, 2003; Abe et al., 2003; Liang et al., 2005; Chen et al., 2006; Jung et al., 2008; Rusconi et al., 2013; Lee et al., 2014). The overexpression of *GmMYB84* and *GmMYB174* in soybean, *GaMYB85*, *GmMYBJ1*, and *GmMYBJ2* in *Arabidopsis*, *OsMYB2* in rice, *MdSIMYB1* in tobacco and apple, and *SbMYB8* in tobacco improved drought tolerance in transgenic plants (Yang et al., 2012; Su et al., 2014; Wang et al., 2014; Su et al., 2015; Wang et al., 2015; Yuan et al., 2015; Butt et al., 2017; Wang et al., 2017b). Until now, the role of MYB116 in plants has not been reported.

The productivity of sweetpotato, *Ipomoea batatas* (L.) Lam., as an important food crop is seriously affected by drought stress. It has been shown that drought tolerance of sweetpotato could be improved through gene engineering (Park et al., 2011; Wang et al., 2016b; Fan et al., 2017; Kang et al., 2018a, Kang et al., 2018b; Kim et al., 2018). However, MYB TFs from sweetpotato have not been functionally characterized to date. In this study, we cloned a novel MYB TF gene named *IbMYB116* from a drought-tolerant sweetpotato line Xushu55-2 and found its overexpression in *Arabidopsis* enhanced drought tolerance through the JA signaling pathway.

## Materials and Methods

### Plant Materials

The drought-tolerant sweetpotato line Xushu55-2 was applied to isolate the *IbMYB116* gene. The expressed sequence tag (EST) was screened from the transcriptome sequencing data of Xushu55-2 (Zhu et al., 2018). The function of *IbMYB116* was identified using *Arabidopsis thaliana* Columbia-0.

### Cloning and Sequence Analysis of *IbMYB116* and Its Promoter

The total RNA from *in vitro*-grown plants of Xushu55-2 was transcribed into the first-strand cDNA (Kang et al., 2018a). The rapid amplification of cDNA ends (RACE) procedure was used to amplify the full-length cDNA of *IbMYB116*. The genomic sequence of *IbMYB116* was amplified from the genomic DNA of *in vitro*-grown Xushu55-2. Its promoter was obtained from the genomic DNA of *in vitro*-grown Xushu55-2 by homology cloning strategy. All of the specific primers are listed in **Supplementary Table S1**. *IbMYB116* was analyzed with online BLAST, ORF Finder, DNAMAN software, Splign tool, and MEGA 7.0, and the *cis*-acting regulatory elements in its promoter region were defined online (Kang et al., 2018b).

### Subcellular Localization of IbMYB116

For subcellular localization, the coding sequence of *IbMYB116* was inserted into the pMDC83 vector. The fusion construct (35S::*IbMYB116::GFP*) and the empty vector (35S::*GFP*) were separately transformed into the EHA105 strain of *Agrobacterium tumefaciens* and further infiltrated the leaves of *Nicotiana benthamiana* (Strasser et al., 2007; Li et al., 2017). After 48 h of growth in a greenhouse, agroinfiltrated leaf sections were imaged at room temperature using a laser scanning confocal microscope with an Argon laser (LSM710, Zeiss, Germany). GFP was excited at 488 nm, and the emitted light was captured at 505–555 nm.

### Transactivation Activity Assay of IbMYB116

The transactivation activity of IbMYB116 was tested in yeast (*Saccharomyces cerevisiae*) (Jiang et al., 2014). The full-length (construct 1) and a series of deletion mutations of *IbMYB116* (constructs 2–9) were fused to the yeast expression vector pGBKT7 (pBD). The expression vector pBD-*IbMYB116*, pGAL4 (positive control), and pBD (negative control) were transferred into the host strain AH109. After the cells were cultured on the SD/-Trp medium, the yeast was further cultured on medium with X-α-Gal (SD/-Trp/-His/X).

### Expression Analysis of *IbMYB116* in Sweetpotato

The transcript levels of *IbMYB116* in the roots, stems, and leaves of the *in vitro*-grown Xushu55-2 plants were analyzed using quantitative real-time PCR (qRT-PCR), the expression levels were normalized to *Ibactin* (AY905538), and the relative expression levels in different tissues were calibrated using the roots (Schmittgen and Livak, 2008; Liu et al., 2014). Furthermore, *in vitro*-grown Xushu55-2 plants were treated in Hoagland solution with H_2_O, 30% PEG6000, and 100 μM MeJA; the plants were sampled 0, 1, 3, 6, 12, and 24 h after treatments and then analyzed for the expression of *IbMYB116.* The expression levels were normalized to *Ibactin* (AY905538), and the relative expression levels in different treatments were calibrated using the plant sampled 0 h after treatment. The specific primers were designed in the nonconserved domain (**Figure S1** and **Supplementary Table S1**).

### Production of the Transgenic *Arabidopsis* Plants

The overexpression vector pC3301-*35S-IbMYB116* was generated by inserting the expression cassette *35S-IbMYB116* into pCAMBIA3301, and then, it was transferred into the *A. tumefaciens* strain GV3101. The transgenic *Arabidopsis* plants were produced and further grown in pots to obtain T_3_ seeds as described by Kang et al. (2018b). The transcript levels of *IbMYB116* in wild-type and transgenic lines were analyzed using qRT-PCR, and the expression levels were normalized to *Atactin* **(NM112764)** (**Supplementary Table S1**). The relative expression levels in transgenic lines were calibrated using the transgenic line with the lowest *IbMYB116* expression.

### Assay for Drought Tolerance

For the germination rate assay, T_3_ and wild-type (WT) seeds sterilized with 2% NaClO for 5 min were sown on 1/2 MS medium with mannitol (0, 100, 200, and 300 mM). Approximately 50 seeds per line were sown for each experiment, and their germination rates were investigated after 3 days. For the root length assay, 5-day-old seedlings of T_3_ and WT formed on 1/2 MS basal medium were vertically cultured on 1/2 MS medium with 0 (as a control) or 300 mM mannitol, and their roots were took out, and the primary root lengths were measured with a ruler after 3 weeks.

Furthermore, the 7-day-old seedlings obtained on 1/2 MS basal medium were transplanted in pots; after 2 weeks, they were stressed by a 4-week drought followed by 2-day rewatering for investigating their phenotypes. The 6-week normal treatment with water was used as a control.

### Expression Analysis of the Related Genes and Measurements of MeJA and H_2_O_2_ Contents

T_3_ and WT plants were pot grown for 2 weeks under drought stress conditions after 2 weeks of normal treatment with water, and they were employed to analyze the expression of the genes involved in JA biosynthesis and signaling pathways as well as the reactive oxygen species (ROS)-scavenging system with qRT-PCR and *Atactin* (NM112764) as an internal control (**Supplementary Table S1**), and the relative expression levels in T_3_ and WT plants were calibrated using WT. Their MeJA and H_2_O_2_ contents were determined using high-performance liquid chromatography (HPLC) and an H_2_O_2_ Assay Kit (Comin Biotechnology Co., Ltd. Suzhou, China), respectively. The 4-week normal treatment with water was used as a control.

### Statistical Analysis

Three biological replicates were performed for all experiments. Difference analysis of data presented as the mean ± SE was done with Student’s *t*-test (two-tailed analysis) using SPSS 20.0 Statistic Program. Significance levels at *P* < 0.05, *P* < 0.01, and *P* < 0.001 were indicated with ∗, ∗∗, and ∗∗∗, respectively.

## Results

### Cloning and Sequence Analysis of *IbMYB116* and Its Promoter

**Figure 1:**
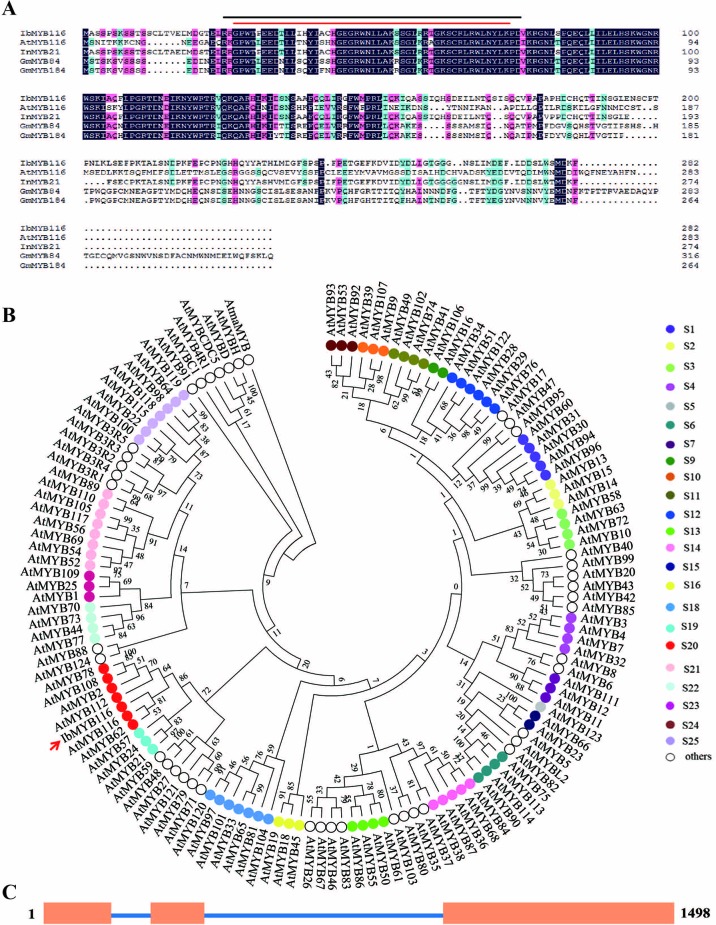
Sequence analysis of IbMYB116. **(A)** Sequence alignment of IbMYB116 with its homologues from other plants. Conserved amino acids are indicated by a dark background. Characteristic regions of MYB are indicated above the IbMYB116 sequence. —, R2 SANT domain; —, R3 SANT domain. **(B)** Phylogenetic analysis of IbMYB116 and MYB transcription factors from *Arabidopsis thaliana*. **(C)** Exon and intron constituents of *IbMYB116*. Exons are represented by boxes and introns by lines.

The *IbMYB116* full-length cDNA was 958 bp in length and contained an 849-bp ORF that encoded a polypeptide of 282 residues with a predicted molecular weight of 31.998 kDa and a *p*I of 6.79. The IbMYB116 protein had two conserved MYB domains that belonged to R2R3-MYB TFs and shared a high-sequence identity with MYB TFs from *Ipomoea nil* (InMYB21, XP_019189643.1, 91.23%), *Glycine max* (GmMYB184, NP_001235837.1, 49.12% and GmMYB84, NP_001235789.1, 43.83%), and *Arabidopsis thaliana* (AtMYB116, AT1G25340.1, 43.24%) (**Figure 1A**). The protein was characterized with a highly conserved DNA-binding sequence at the N-terminus (**Figure 1A**). Phylogenetic analysis along with 125 *Arabidopsis* R2R3-MYB TFs belonging to 24 groups (Dubos et al., 2010) revealed that IbMYB116 belonged to group S20 of the MYB family and had a close relationship with AtMYB116 (At1G25340.1) (**Figure 1B**). The genomic DNA of *IbMYB116* was 1,498 bp with three exons and two introns (**Figure 1C**). Its promoter region (∼1,455 bp) contained the *cis*-acting regulatory elements associated with stresses and phytohormones, including LTR, HSE, MBS, GARE-motif, CGTCA-motif, TGACG-motif, P-box, and SARE (**Figure S2**).

### Subcellular Location of IbMYB116

**Figure 2:**
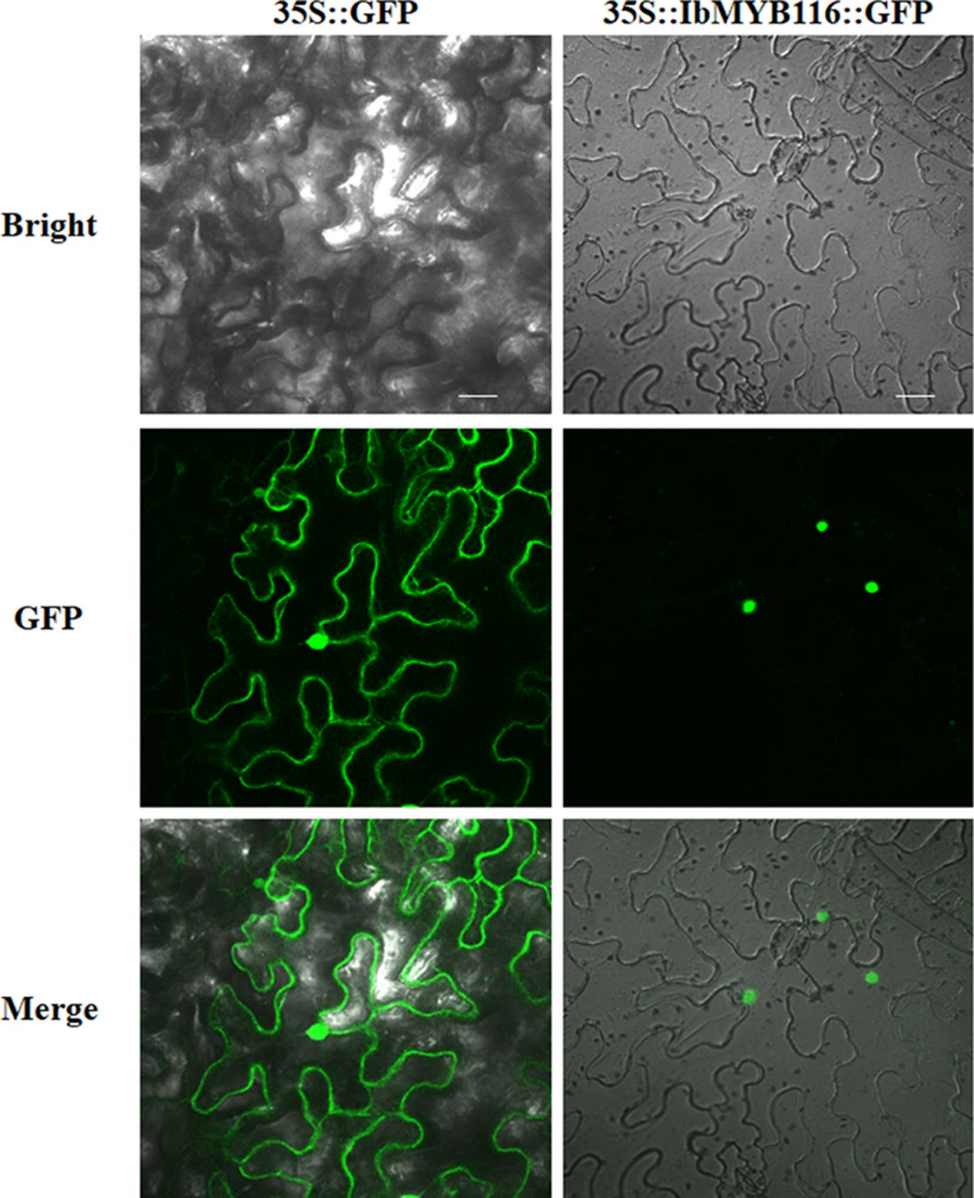
Subcellular localization of IbMYB116 in tobacco leaf hypodermal cells. Confocal scanning microscopic images show localization of IbMYB116-GFP fusion proteins to nuclei in the right column vs. GFP as the control in the left column. Bars = 20 µm.

IbMYB116-GFP showed fluorescence in the nuclei of *Nicotiana benthamiana* leaf hypodermal cells, while the fluorescence of GFP was distributed in the entire cell (**Figure 2**). These results clearly indicated that IbMYB116 is a nuclear protein.

### Transactivation Activity of IbMYB116 in Yeast

**Figure 3:**
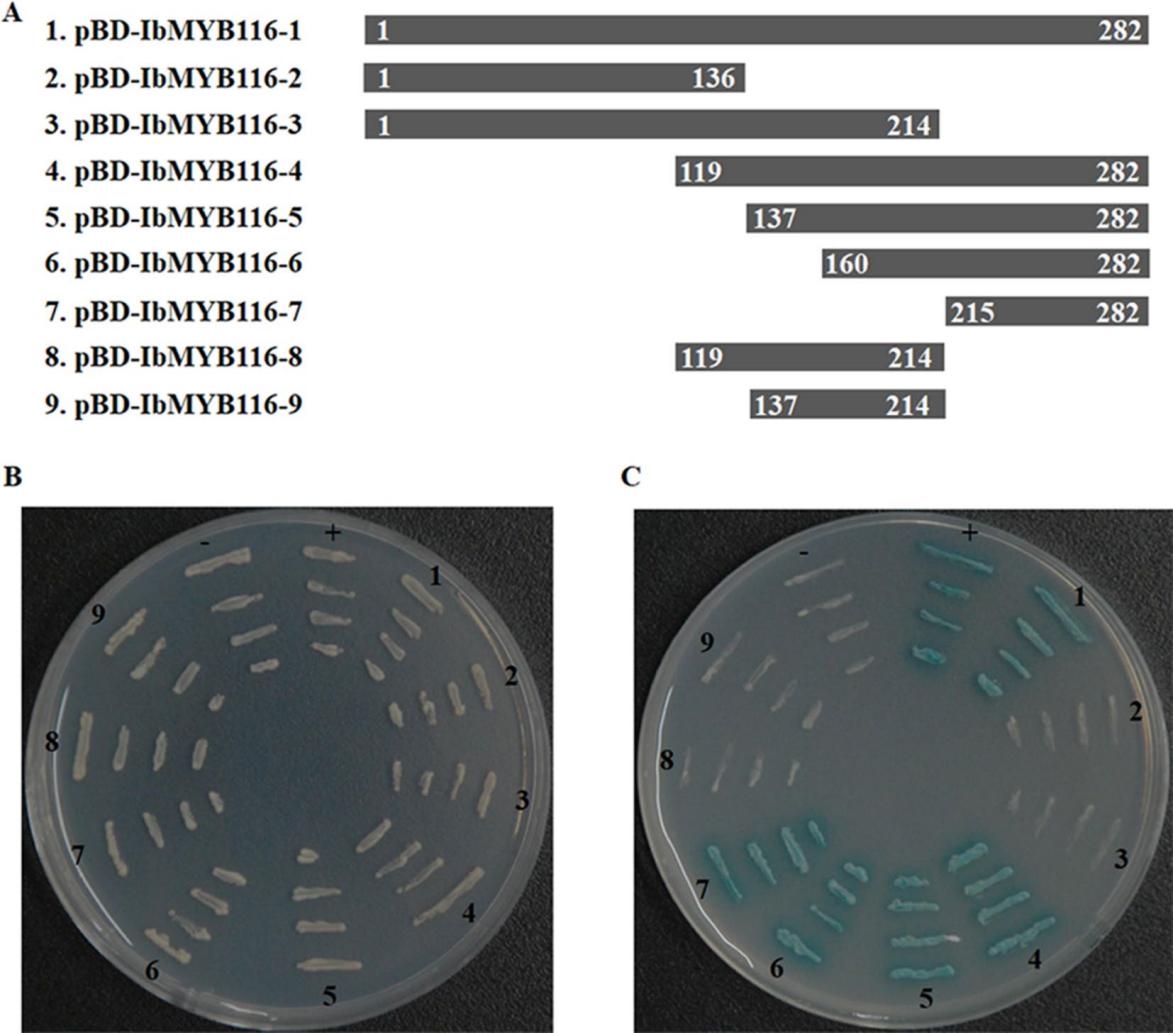
Transactivation activity assay of IbMYB116 in yeast. **(A)** Diagram showing a full-length construct (construct 1) and a series of deletion mutations of *IbMYB116* (constructs 2–9). **(B)** The transformed yeast cells harboring different expression vectors (constructs 1–9) were drawn onto SD/-Trp medium. pBD (**–**) and pGAL4 (**+**) were used as negative and positive controls, respectively. **(C)** The transformed yeast cells harboring different expression vectors (constructs 1–9) were drawn onto SD/-Trp/-His medium supplemented with X-α-Gal. pBD (**−**) and pGAL4 (**+**) were used as negative and positive controls, respectively.

All of the transformed yeast cells grew well on the SD/-Trp medium, which indicated that expression vectors (pBD-*IbMYB116*-1, -2, -3, -4, -5, -6, -7, -8, -9; **Figure 3A**) had been successfully transferred into yeast cells (**Figure 3B**). The yeast cells harboring pBD-*IbMYB116*-1, -4, -5, -6, -7, and pGAL4 (positive control) grew well and turned blue, but the cells with pBD-*IbMYB116*-2, -3, -8, -9, and pBD (negative control) failed to grow on medium with X-α-Gal (SD/-Trp/-His/X) (**Figure 3C**). These results demonstrated that IbMYB116 contained the transactivation activity domain at the region of amino acids 215–282.

### Expression of *IbMYB116* in Sweetpotato

**Figure 4:**
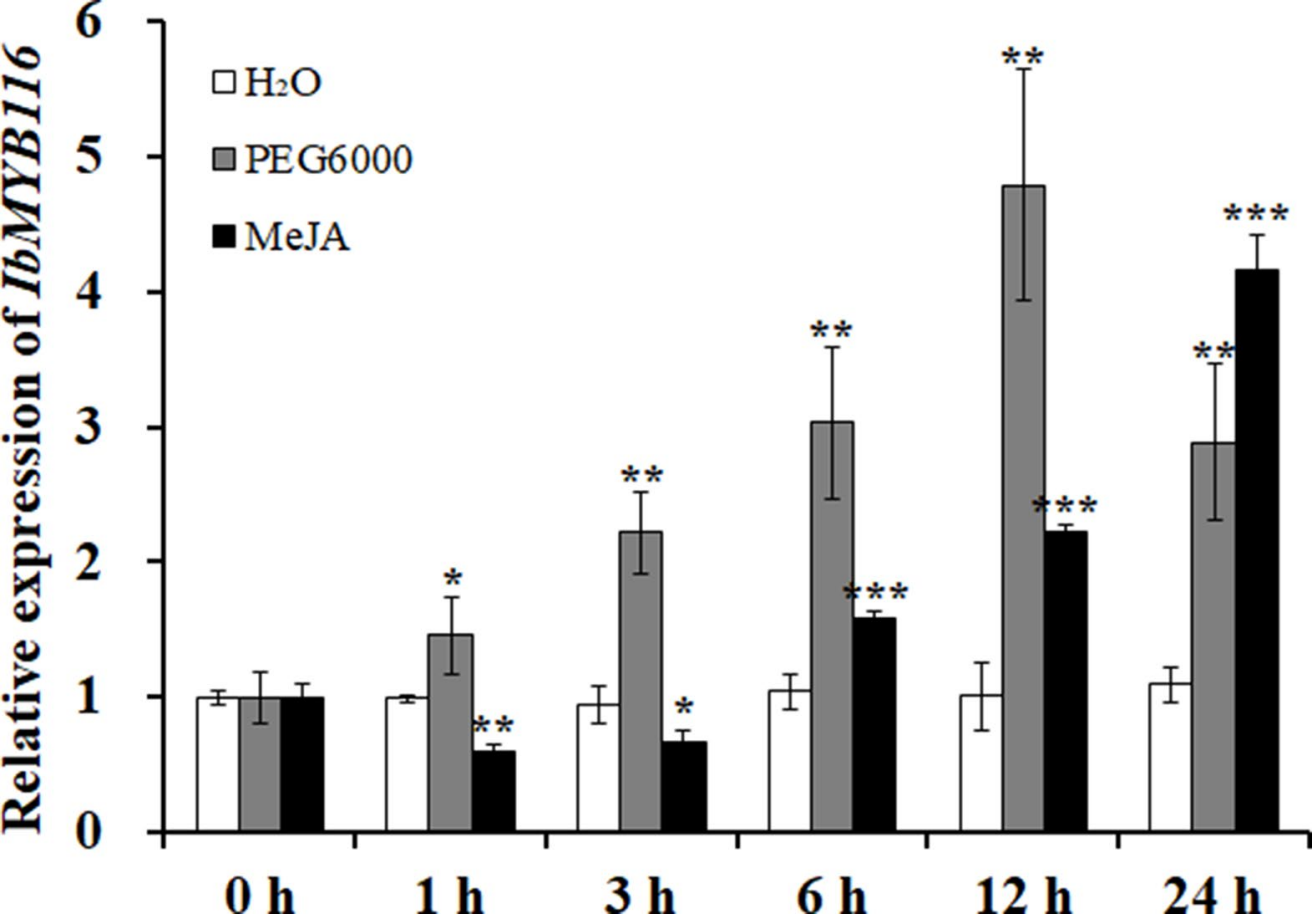
Expression analysis of *IbMYB116* in the *in vitro*-grown plants of Xushu55-2 after different times (h) in response to H_2_O, 30% PEG6000, and 100 µM MeJA, respectively. Data are presented as the means ± SE (*n* = 3). *, ** and *** indicate significant differences from WT at *P* <0.05, *P* <0.01, and *P* <0.001, respectively, according to the Student’s *t*-test.

A significantly higher expression level of *IbMYB116* was found in the leaves of Xushu55-2 than that in the roots and stems (**Figure S3**). Its expression in Xushu55-2 was strongly induced by 30% PEG6000 and 100 μM MeJA, and it reached the highest level at 12 h (4.78-fold) and 24 h (4.15-fold), respectively (**Figure 4**).

### Production of the Transgenic *Arabidopsis* Plants

The transgenic *Arabidopsis* plants were obtained using the protocols of Kang et al. (2018b). Eight transgenic plants named L1, L2, …, L8 were randomly selected to analyze the expression level of *IbMYB116* with qRT-PCR (**Figure S1**). The results showed that all of the transgenic plants exhibited significantly higher expression levels of *IbMYB116* than the WT (**Figure S4**).

### Enhanced Drought Tolerance

**Figure 5:**
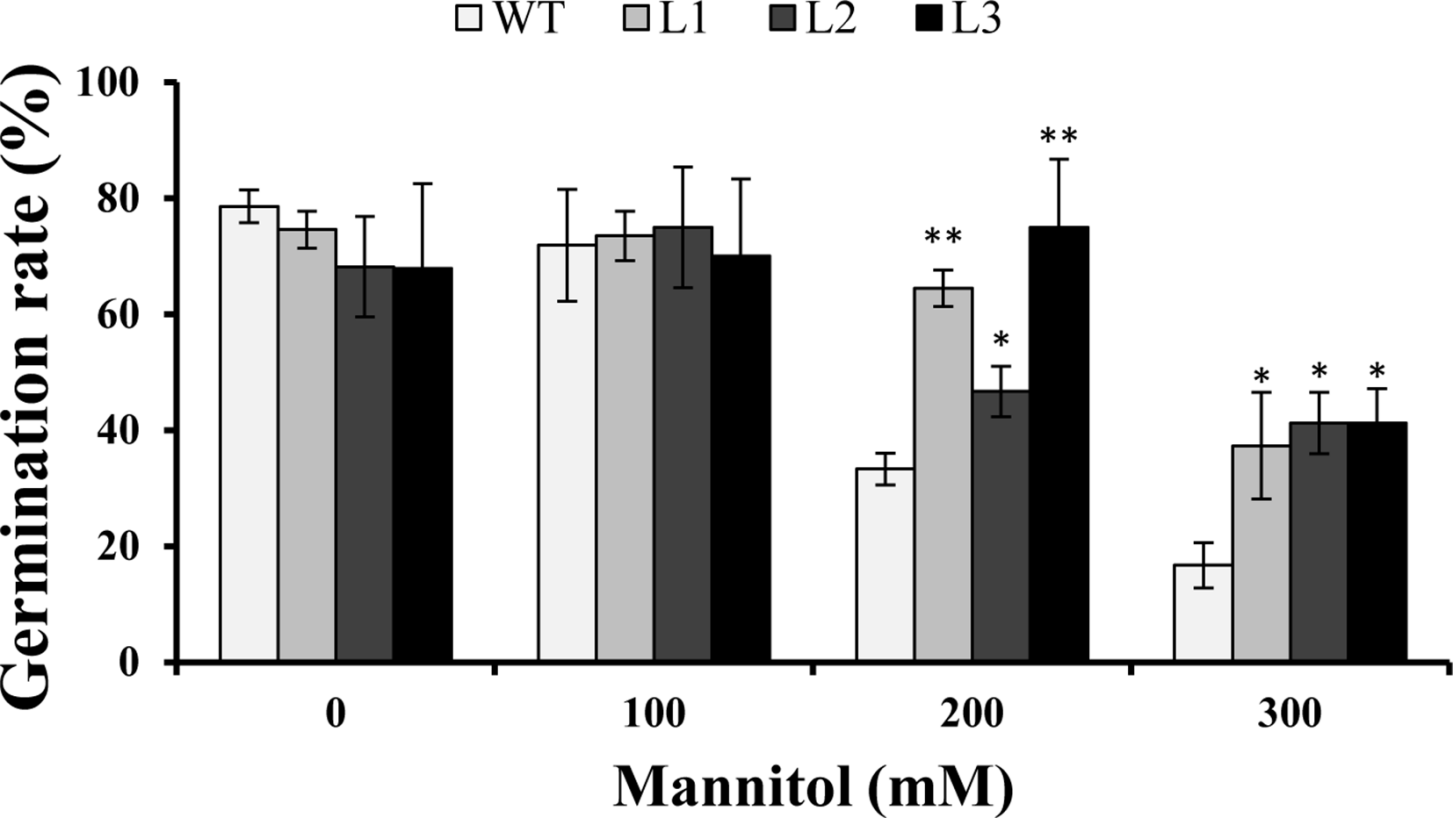
Germination assay of the transgenic Arabidopsis and WT seeds sown for 3 days on 1/2 MS medium with 0, 100, 200, and 300 mM mannitol, respectively. * and ** indicate significant differences from WT at *P* < 0.05 and *P* < 0.01, respectively, according to the Student’s *t*-test.

**Figure 6:**
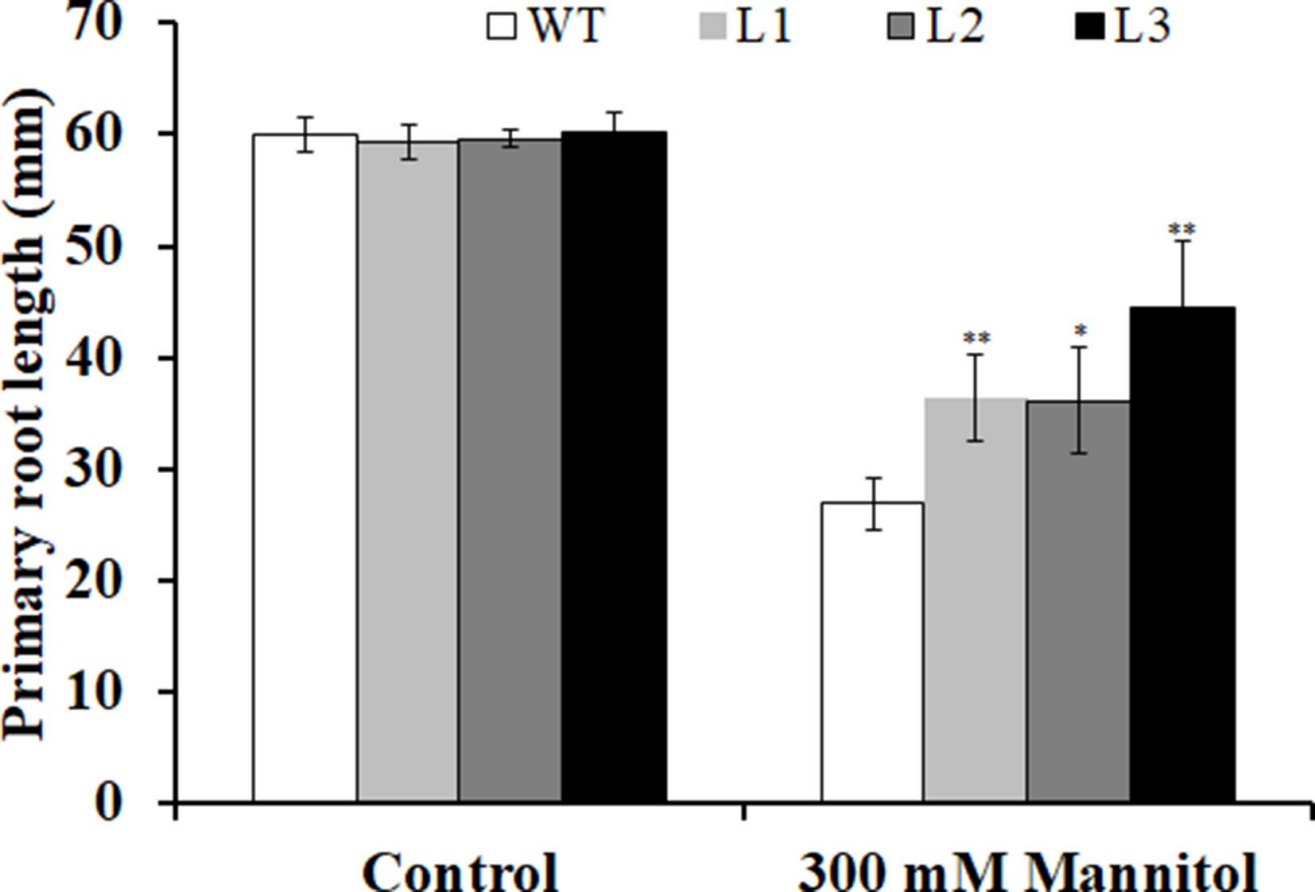
Primary root lengths of the transgenic *Arabidopsis* and WT seedlings cultured for 3 weeks on 1/2 MS medium without (control) and with 300 mM mannitol, respectively. All the plants were grown in a standard square plate of 12 × 12 cm. Data are presented as the mean ± SE (*n* = 3). * and ** indicate significant differences from WT at *P* < 0.05 and *P* < 0.01, respectively, according to the Student’s *t*-test.

Three transgenic *Arabidopsis* plants (L1, L2, and L3) with high expression levels of *IbMYB116* were selected for investigating their drought tolerance. The transgenic and WT plants showed no differences in germination rates on 1/2 MS media with 0 (control) and 100 mM mannitol (**Figure 5**). However, the transgenic plants provided significantly higher germination rates than the WT under 200 and 300 mM mannitol stress conditions (**Figure 5**). The transgenic and WT plants showed similar growth on 1/2 MS media without mannitol, but the transgenic plants formed significantly longer roots than the WT at the level of 300 mM mannitol (**Figure 6**).

**Figure 7:**
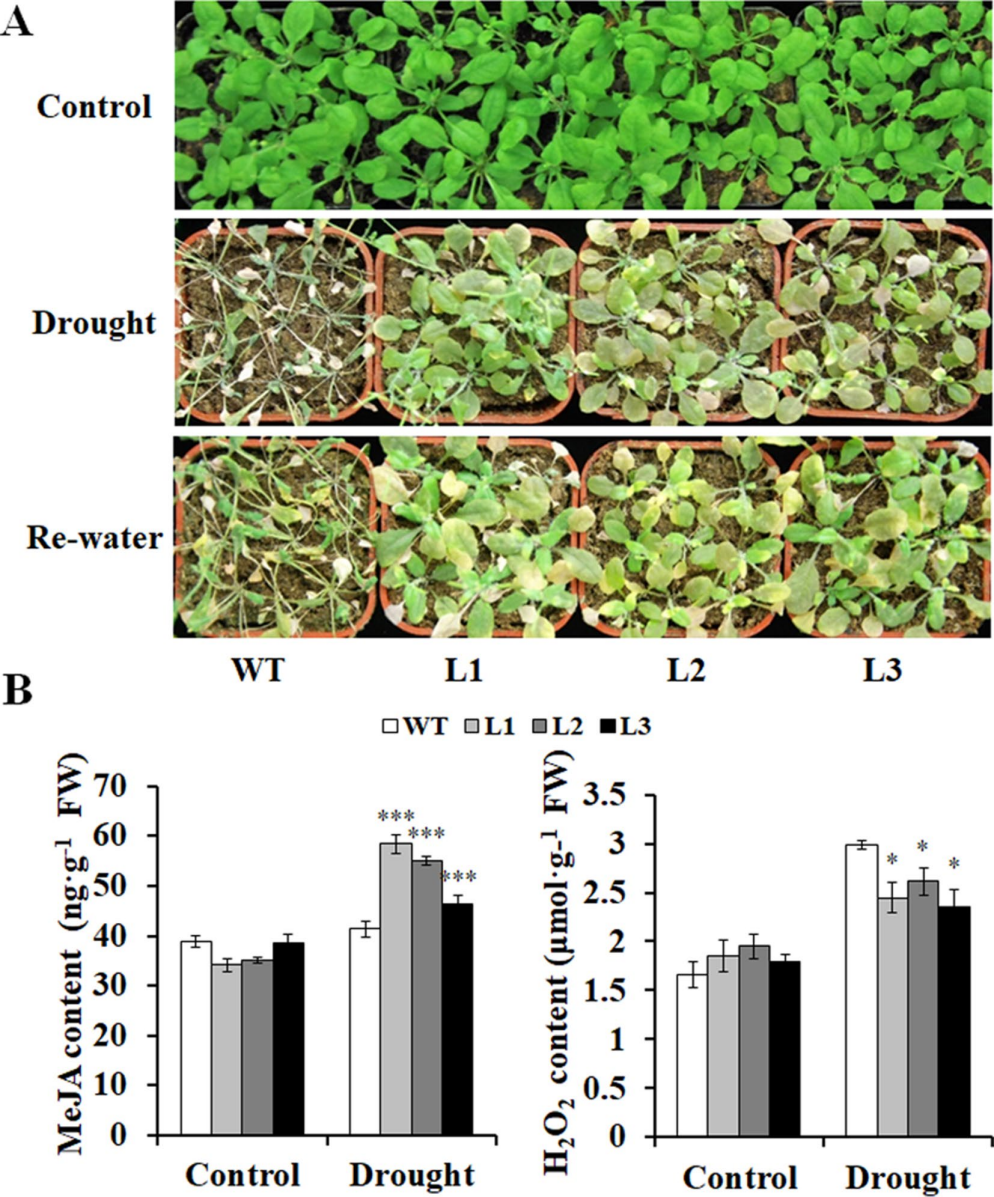
Responses of the transgenic *Arabidopsis* plants and WT to drought stress. **(A)** Phenotypes of the transgenic plants vs. WT stressed by a 4-week drought followed by 2 days rewatering after 2 weeks of normal treatment with water. The 6-week normal treatment was used as a control. **(B)** Contents of MeJA and H_2_O_2_ in the leaves of the transgenic and WT plants grown for 4 weeks under normal treatment (control) and 2 weeks under drought stress after 2 weeks of normal treatment, respectively. *, **, and *** indicate significant differences from WT at *P* < 0.05, *P* < 0.01, and *P* < 0.001, respectively, according to the Student’s *t*-test.

Furthermore, the transgenic and WT plants grown in pots had no differences in growth without drought stress (**Figure 7A**). However, after being stressed by drought, the transgenic plants exhibited better growth, increased MeJA content, and decreased H_2_O_2_ content compared with the WT (**Figures 7A, B**). These results indicated that the transgenic *Arabidopsis* plants had significantly enhanced drought tolerance compared with the WT.

### Expression of the Related Genes

**Figure 8:**
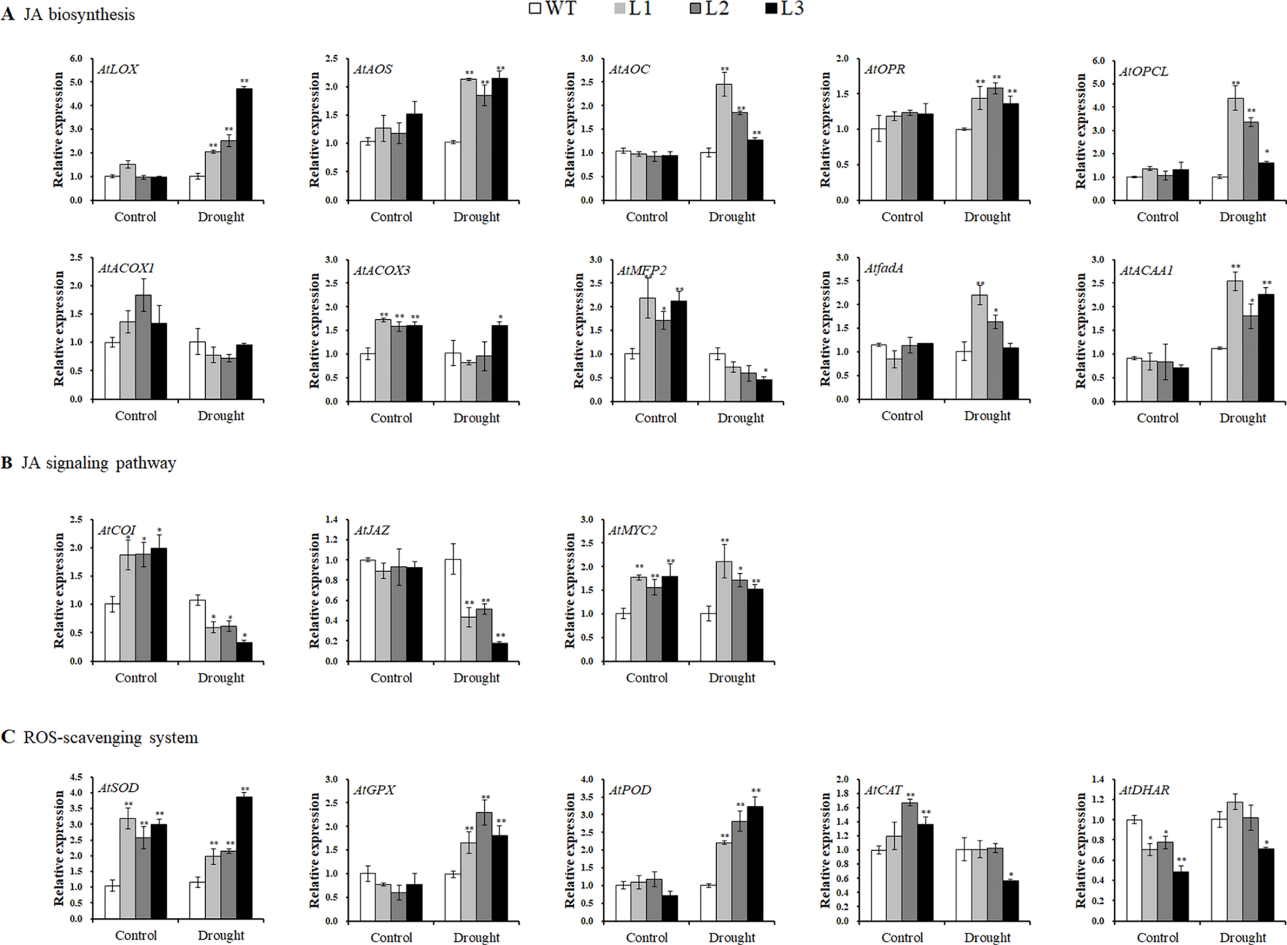
Transcript levels of the related genes in the leaves of the transgenic *Arabidopsis* and WT plants pot grown for 4 weeks under normal treatment with water (control) and 2 weeks under drought stress after 2 weeks of normal treatment, respectively. **(A)** Transcript levels of JA biosynthesis genes. **(B)** Transcript levels of JA signaling pathway genes. **(C)** Transcript levels of ROS-scavenging system genes. * and ** indicate significant differences from WT at *P* < 0.05 and *P* < 0.01, respectively, according to the Student’s *t*-test.

Systematic upregulation of the JA biosynthesis key enzyme genes *AtLOX*, *AtAOS*, *AtAOC*, *AtOPR*, *AtOPCL*, *AtACOX3*, *AtfadA*, and *AtACAA1*, except for *AtACOX1* (no change) and *AtMFP2* (downregulated), was found in the transgenic *Arabidopsis* plants under drought stress conditions (**Figure 8A**). In the JA signaling pathway, *AtCOI1* and *AtJAZ* were downregulated, but *AtMYC2* was upregulated under drought stress conditions (**Figure 8B**). The genes encoding ROS scavenging enzymes superoxide dismutase (SOD), glutathione peroxidase (GPX), and peroxidase (POD) were also upregulated, and both *AtCAT* and *AtDHAR* apparently had not changed (**Figure 8C**).

## Discussion

### *IbMYB116* Enhances Drought Tolerance

It has been shown that several MYB TFs play crucial roles in the response to drought stress as positive stress response TFs (Rusconi et al., 2013; Lee et al., 2014; Yuan et al., 2015; Butt et al., 2017; Wang et al., 2017b). To date, the role of MYB116 in plants is still unknown. In this study, we cloned the *IbMYB116* gene from the drought-tolerant sweetpotato line Xushu55-2 and found that the IbMYB116 protein belonged to R2R3-MYB TFs (**Figure 1**) (Jia et al., 2004; He et al., 2016). This gene was strongly induced by PEG6000 and MeJA, and its overexpression significantly improved the drought tolerance of the transgenic *Arabidopsis* plants (**Figures 4** **–** **7**). This study reveals, for the first time, the crucial role of *IbMYB116* in the drought tolerance of plants.

### *IbMYB116* Upregulates JA Signaling Pathway Genes

JAs are naturally occurring signaling compounds that regulate plant responses to abiotic stresses, such as drought, salt, and ozone exposure (Rojo et al., 2003; Ahmad et al., 2016; Dhakarey et al., 2017). Rohwer and Erwin (2008) found that JA and JA-induced protein levels were increased in sorbitol-treated barley leaves, which confirmed the role of JAs in osmotic stress. MeJA enhanced drought tolerance by improving the water status of wheat plants (Ma et al., 2004) or increasing antioxidant activities in soybean plants (Anjum et al., 2011). In *Arabidopsis*, four 13-LOX forms (AtLOX2, 3, 4, and 6) contributed to JA formation in the stress response (Caldelari et al., 2011; Chauvin et al., 2013). The upregulation of *OsLOX*, *OsAOS2*, and *OsOPR7* led to massive accumulation of endogenous JA in the rice *rim1* mutant (Yoshii et al., 2010). The tolerance to drought as well as osmotic and salinity changes were increased in the *Arabidopsis* plants overexpressing *CaLOX1* from pepper (Lim et al., 2015). The *TaAOC1*-overexpressing wheat and *Arabidopsis* plants accumulated more JA and exhibited enhanced tolerance to changes in salinity, which indicated that JA is related to the plant salinity response (Zhao et al., 2014). The expression of *OsOPR7* was induced by drought stress and wounding, and the increase in *OsOPR7* expression led to an elevated endogenous JA level (Tani et al., 2008).

The JA signaling pathway plays an important role in regulating the plant response to drought stress (Liu et al., 2015; Ahmad et al., 2016). As components of the core JA signaling pathway, COI1 participates in removing repressors of JA transduction (Xie et al., 1998; Ahmad et al., 2016); JAZs are transcription repressors of JA-responsive genes (Chini et al., 2007), and MYC2 acts as a positive regulator of JA signals (Zhang et al., 2015b). The overexpression of *AtMYC2* in *Arabidopsis* improved osmotic stress tolerance (Abe et al., 2003). The *OsMYC2*-overexpressing rice exhibited increased resistance against Xoo (Uji et al., 2016).

**Figure 9:**
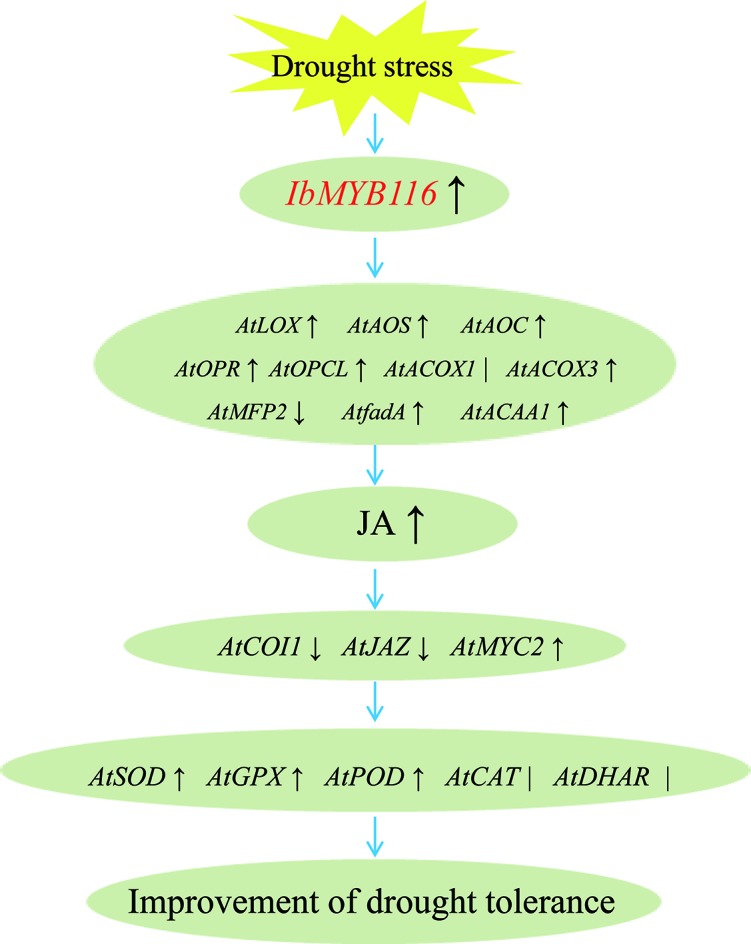
Diagram showing a proposed model for the regulation of *IbMYB116* in drought tolerance for transgenic *Arabidopsis*. ↑, ↓and ∣ indicate upregulation, downregulation, and no obvious change of expression for genes coding these enzymes, respectively.

Our results showed that overexpression of *IbMYB116* in *Arabidopsis* systematically upregulated the JA biosynthesis genes, including *AtLOX*, *AtAOS*, *AtAOC*, *AtOPR*, *AtOPCL*, *AtCOX3*, *AtfadA*, and *AtACAA1*, and the MeJA content increased under drought stress conditions (**Figures 7**, **8**). *AtCOI1* and *AtJAZ* were downregulated, and *AtMYC2* was upregulated in the transgenic *Arabidopsis* plants under drought stress conditions (**Figure 8**). These findings suggest that *IbMYB116* enhances drought tolerance through the JA signaling pathway in transgenic *Arabidopsis* (**Figure 9**).

### *IbMYB116* Activates the ROS-Scavenging System

In plants, ROS cause damage to the structure and function of biomolecules, which leads to oxidative stress. H_2_O_2_, as one of the prominent ROS, is considered to be an important signaling molecule in plant cells (Wang et al., 2017a). The ROS-scavenging system can detoxify ROS to reduce oxidative damage in plant cells (Gill and Tuteja, 2010; Liu et al., 2014). It has been shown that JA can activate the ROS-scavenging system in plants. The overexpression of *CaLOX1* in *Arabidopsis* systematically upregulated the ROS-scavenging system genes, which resulted in the reduced H_2_O_2_ level under drought and salt stress conditions (Lim et al., 2015). The JA-deficient tomato mutant *def-1* showed higher ROS levels than the WT under salt stress conditions (Abouelsaad and Renault, 2018). JA influenced oxidative stress through its direct effect on the activities of ROS-scavenging enzymes in raspberry and strawberry (Wang, 1999; Ghasemnezhad and Javaherdashti, 2008). Reduced levels of JA led to more GLN18:3-induced ROS production in *Arabidopsis* and tomato (Block et al., 2018).

The results of this study demonstrated that ROS-scavenging system genes, including *AtSOD*, *AtGPX*, and *AtPOD*, were upregulated and that the H_2_O_2_ level was decreased in the *IbMYB116*-overexpressing *Arabidopsis* plants under drought stress conditions (**Figures 7**, **8**). These results suggest that *IbMYB116* might enhance drought tolerance by activating the ROS-scavenging system through the JA signaling pathway in transgenic *Arabidopsis* (**Figure 9**).

## Conclusion

We found a crucial role for *IbMYB116* in the drought tolerance of plants. The results suggest that the overexpression of *IbMYB116* might enhance drought tolerance by activating the ROS-scavenging system through the JA signaling pathway in transgenic *Arabidopsis*. This gene has the potential to be used for improving the drought tolerance of sweetpotato and other plants.

## Author Contributions

QL and YZ conceived and designed the experiments. YZ and HZu performed the experiments. YZ, SH, and HZa analyzed the data. QL, NZ, SX, and ZW contributed reagents, materials, and analysis tools. QL and YZ wrote the paper. All authors read and approved the final manuscript.

## Funding

This work was supported by the National Natural Science Foundation of China (31461143017) and the China Agriculture Research System (CARS-10, Sweetpotato).

## Conflict of Interest Statement

The authors declare that the research was conducted in the absence of any commercial or financial relationships that could be construed as a potential conflict of interest.
