# Supplementary material for: A Novel Sweetpotato Transcription Factor Gene IbMYB116 Enhances Drought Tolerance in Transgenic Arabidopsis
Source: Front Plant Sci. 2019 Aug 15;10:1025. doi: 10.3389/fpls.2019.01025 (PMC6704235; doi:10.3389/fpls.2019.01025)
Supplement: Supplementary file 1 [file Image_1.pdf]

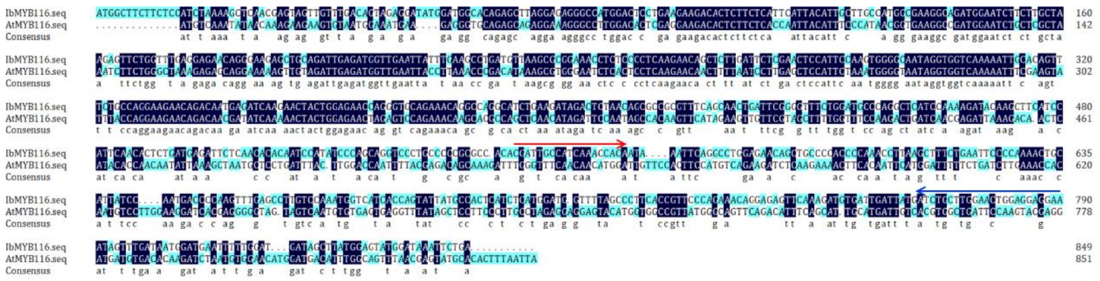

FIGURE S1 | Sequence alignment of *IbMYB116* with *AtMYB116*. → and ← indicate forward primer (IbMYB116-F: CGATTGCCATCAAACCACA) and reverse primer (IbMYB116-R: CTCCTCCAGTTCCAAGCAGAT).

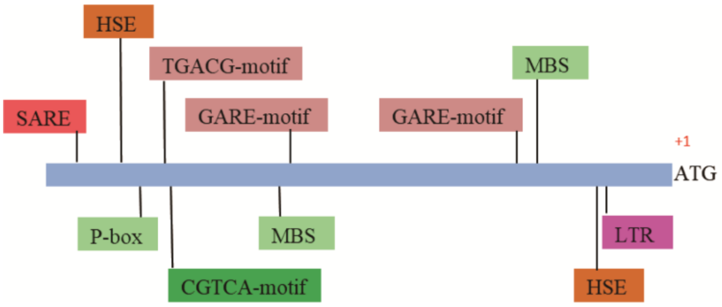

FIGURE S2 | The promoter of *IbMYB116* showing different *cis*-acting regulatory elements associated with abiotic stresses.

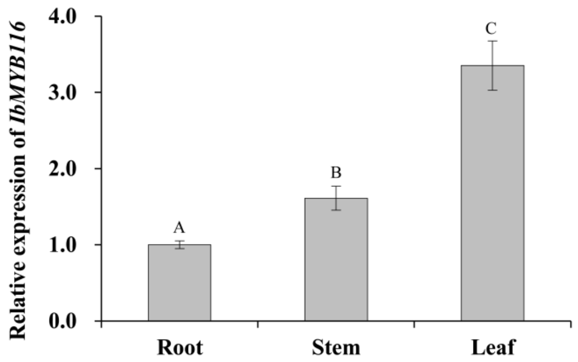

FIGURE S3 | Expression analysis of *IbMYB116* in root, stem and leaf tissues of *in vitro*-grown Xushu55-2. Data are presented as the means ± SE (n = 3). Different capital letters indicate a significant difference at P <0.01 according to the Student's *t*-test.

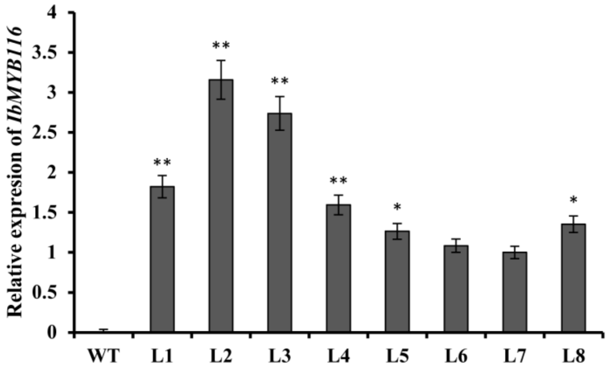

FIGURE S4 | Expression analysis of *IbMYB116* in the transgenic *Arabidopsis* plants. *Atactin* (NM112764) was used as an internal control and the relative expression levels were calibrated using the transgenic line (L7) with the lowest expression. Data are presented as the means ± SE (n = 3). * and ** indicate significant differences from L7 at P <0.05 and P <0.01, respectively, according to the Student's *t*-test.
