# Supplementary material for: A Novel Sweetpotato Transcription Factor Gene IbMYB116 Enhances Drought Tolerance in Transgenic Arabidopsis
Source: Front Plant Sci. 2019 Aug 15;10:1025. doi: 10.3389/fpls.2019.01025 (PMC6704235; doi:10.3389/fpls.2019.01025)
Supplement: Supplementary file 2 [file Table_1.docx]

| **Table S1** Primers used in this study | |
| --- | --- |
| Primer name | Primer sequence (5’-3’) |
| Primers for 5’/3’ RACE | |
| 5GSP1 | CCAGTAGTTCTTGATCTCATTGTCT |
| 5GSP2 | CTGAAACGCGGCGCTGTTAGAGTCT |
| 5GSP3 | CCTGCTGGGATATGGATTGTGTGTT |
| 3GSP1 | AGCTTTCTGAATTCCCCAAAACTGC |
| 3GSP2 | GTTTTAGCCCTTCACCGTTCCCAGA |
| Primers for 5^’^-promoter region |  |
| GW1 | GCCCTCTCCTAAGCTCTGT |
| GW2 | CAAGAAGATTCCATCTGCCTTC |
| Primers for constructing vectors | |
| OS-F- *Bgl*Ⅱ | GAAGATCTATGGCTTCTTCTCCATCTAAAAGCT |
| OS-R-*Pml*Ⅰ | GCCACGTGTCAGAATTTATCCATACTCCATAAG |
| 83S-F-*Spe*I | GACTAGTATGGCTTCTTCTCCATCTAAAAGCT |
| 83S-R-*Asc*I | GGCGCGCCAGAATTTATCCATACTCCATAAG |
| pBD-1-F-*Nde*I | GGAATTCCATATGATGGCTTCTTCTCCATCTAAAAG |
| pBD-1-R-*Sal*I | GCGTCGACTCAGAATTTATCCATACTCCATAAG |
| pBD-2-F-*Nde*I | GGAATTCCATATGATGGCTTCTTCTCCATCTAAAAG |
| pBD-2-R-*Sal*I | GCGTCGACGTTAGAGTCTATCTTCAGATGCCTG |
| pBD-3-F-*Nde*I | GGAATTCCATATGATGGCTTCTTCTCCATCTAAAAG |
| pBD-3-R-*Sal*I | GCGTCGACGGATAATGCAGTTTTGGGG |
| pBD-4-F-*Nde*I | GGAATTCCATATGTACTGGAGAACCAGGGTGC |
| pBD-4-R-*Sal*I | GCGTCGACTCAGAATTTATCCATACTCCATAAG |
| pBD-5-F-*Nde*I | GGAATTCCATATGAGCGCCGCGTTTCAG |
| pBD-5-R-*Sal*I | GCGTCGACTCAGAATTTATCCATACTCCATAAG |
| pBD-6-F-*Nde*I | GGAATTCCATATGTCCATTCAACACTCTGATGAG |
| pBD-6-R-*Sal*I | GCGTCGACTCAGAATTTATCCATACTCCATAAG |
| pBD-7-F-*Nde*I | GGAATTCCATATGTTGTCCAAATGGTCATCACC |
| pBD-7-R-*Sal*I | GCGTCGACTCAGAATTTATCCATACTCCATAAG |
| pBD-8-F-*Nde*I | GGAATTCCATATGTACTGGAGAACCAGGGTGC |
| pBD-8-R-*Sal*I | GCGTCGACGGATAATGCAGTTTTGGGG |
| pBD-9-F-*Nde*I | GGAATTCCATATGAGCGCCGCGTTTCAG |
| pBD-9-R-*Sal*I | GCGTCGACGGATAATGCAGTTTTGGGG |
| Pro-F | CTTATACGGTAAACGGGTCG |
| Pro-R | GCTTGATGAAATGGCAGGA |
| Primers for identifying transformants | |
| 35S-F | GAACTCGCCGTAAAGACTGG |
| *IbMYB116*-R | TCAGAATTTATCCATACTCCATAAG |
| Primers for qRT-PCR | |
| *Ibactin*-F | AGCAGCATGAAGATTAAGGTTGTAGCAC |
| *Ibactin*-R | TGGAAAATTAGAAGCACTTCCTGTGAAC |
| *IbMYB116*-F | CGATTGCCATCAAACCACA |
| *IbMYB116*-R | CTCCTCCAGTTCCAAGCAGAT |
| *Atactin*-F | GCACCCTGTTCTTCTTACCGA |
| *Atactin*-R | AGTAAGGTCACGTCCAGCAAGG |
| *AtLOX*-F | CAAACCTCAGAAGACGATGTAAGG |
| *AtLOX*-R | GACCTCTCGACCAAGTTATGCC |
| *AtAOS*-F | CGATTTCTCTCCACCCAAAAAC |
| *AtAOS*-R | GGTCTTTGATTGGTCCTACGATT |
| *AtAOC*-F | TCAGAACTTGGGAAATACCGAA |
| *AtAOC*-R | TAAGAATTTTTGGGCTGTGTCG |
| *AtOPR*-F | CTACTGTCATGTGATTGAAGCG |
| *AtOPR*-R | AAAAGTCCCCTTAAACGCTTTC |
| *AtOPCL*-F | CTACTGTCATGTGATTGAAGCG |
| *AtOPCL*-R | AAAAGTCCCCTTAAACGCTTTC |
| *AtACOX1*-F | GAGGATATGAAGATCGTCTGGG |
| *AtACOX1*-R | TCATTGAGACGAAGCTCGATAA |
| *AtACOX3*-F | GATCACAATGAAACGGATCTGG |
| *AtACOX3*-R | AGACGGAGTGATCATAAATCCC |
| *AtMFP2*-F | CCGTCAATTCTCTATCCTTCGA |
| *AtMFP2*-R | AGTCGGTTATGATGTCAATCGA |
| *AtfadA*-F | TTCACAGTTCTTCCACTAGACC |
| *AtfadA*-R | GTGAAACAAGTAACCCCATGTC |
| *AtACAA1*-F | CGGTTTAATGTTTCAAGGGAGG |
| *AtACAA1*-R | AGCAGTTGTGGTTCCGTCTTC |
| *AtCOI1-*F | TTGCTGAACAAGTTATGGAAGC |
| *AtCOI1*-R | CTGCTGCAGAGTCATTCTACTA |
| *AtJAZ1*-R | CGGGCAAGTGATTGTATTCAAT |
| *AtJAZ1*-R | AGGAACTTGGTTTGCGATAGTA |
| *AtMYC2*-F | CGGATCAGGAGTACAGGAAAAA |
| *AtMYC2*-R | GAAAAACCATTCCGTATCCGTC |
| *AtSOD*-F | ATGAGAAGTTCTATGAAGAG |
| *AtSOD*-R | GTCTTTATGTAATCTGGT |
| *AtGPX*-F | ATGGCGACGAAGGAACCAG |
| *AtGPX*-R | ATCGCCGAAGATTCCCCATTT |
| *AtPOD*-F | TCCGGGAGCACACCATTGG |
| *AtPOD*-R | TGGTCGGAATTCAACAG |
| *AtCAT*-F | GCAACTACCCCCGAGTGGAAA |
| *AtCAT*-R | TGTTCAGAACCAAGCGACCA |
| *AtDHAR*-F | ATGGTCCTTTTATCGCCGGG |
| *AtDHAR*-R | GCCCATCCAGAGATCACACA |
